# SADS-CoV nsp1 inhibits the STAT1 phosphorylation by promoting K11/K48-linked polyubiquitination of JAK1 and blocks the STAT1 acetylation by degrading CBP

**DOI:** 10.1016/j.jbc.2024.105779

**Published:** 2024-02-21

**Authors:** Yingjie Xiang, Chunxiao Mou, Liqi Zhu, Ziyan Wang, Kaichuang Shi, Wenbin Bao, Jiarui Li, Xiang Chen, Zhenhai Chen

**Affiliations:** 1 College of Veterinary Medicine, Yangzhou University, Yangzhou, China; 2 Jiangsu Co-Innovation Center for Prevention and Control of Important Animal Infectious Diseases and Zoonoses, Yangzhou University, Yangzhou, China; 3 Jiangsu Key Laboratory of Zoonosis, Yangzhou University, Yangzhou, China; 4 Guangxi Center for Animal Disease Control and Prevention, Nanning, Guangxi, China; 5 Key Laboratory for Animal Genetics, Breeding, Reproduction and Molecular Design of Jiangsu Province, College of Animal Science and Technology, Yangzhou University, Yangzhou, China; 6 Joint International Research Laboratory of Agriculture and Agri-Product Safety, The Ministry of Education of China, Yangzhou University, Yangzhou, China

**Keywords:** swine acute diarrhea syndrome coronavirus, nonstructural protein 1, signal transducer and activator of transcription 1, Janus kinase 1, ubiquitination

## Abstract

The newly discovered zoonotic coronavirus swine acute diarrhea syndrome coronavirus (SADS-CoV) causes acute diarrhea, vomiting, dehydration, and high mortality rates in newborn piglets. Although SADS-CoV uses different strategies to evade the host’s innate immune system, the specific mechanism(s) by which it blocks the interferon (IFN) response remains unidentified. In this study, the potential of SADS-CoV nonstructural proteins (nsp) to inhibit the IFN response was detected. The results determined that nsp1 was a potent antagonist of IFN response. SADS-CoV nsp1 efficiently inhibited signal transducer and activator of transcription 1 (STAT1) phosphorylation by inducing Janus kinase 1 (JAK1) degradation. Subsequent research revealed that nsp1 induced JAK1 polyubiquitination through K11 and K48 linkages, leading to JAK1 degradation *via* the ubiquitin–proteasome pathway. Furthermore, SADS-CoV nsp1 induced CREB-binding protein degradation to inhibit IFN-stimulated gene production and STAT1 acetylation, thereby inhibiting STAT1 dephosphorylation and blocking STAT1 transport out of the nucleus to receive antiviral signaling. In summary, the results revealed the novel mechanisms by which SADS-CoV nsp1 blocks the JAK–STAT signaling pathway *via* the ubiquitin–proteasome pathway. This study yielded valuable findings on the specific mechanism of coronavirus nsp1 in inhibiting the JAK–STAT signaling pathway and the strategies of SADS-CoV in evading the host’s innate immune system.

Coronaviruses have significantly challenged worldwide public health in the last 20 years. Coronaviruses are frequently present in animal and human groups and can cause economic disruption and catastrophic loss of life (1). In February 2017, the newly discovered swine acute diarrhea syndrome coronavirus (SADS-CoV) was first reported in southern China. SADS-CoV infection leads to acute diarrhea, vomiting, and high mortality rates among young piglets (particularly those <7 days old) but only causes mild or asymptomatic infections in adult swine (2, 3).

SADS-CoV is a positive-sense, single-stranded enveloped RNA virus with a genome size of approximately 27 kb, which encodes 16 nonstructural proteins (nsp), four structural proteins, and three accessory proteins (4, 5). SADS-CoV is believed to have originated from bats before crossing species to infect swine (6, 7). Earlier studies demonstrated that SADS-CoV displays a wide range of cell tropisms and replicates in different vertebrate cell types, indicating its potential zoonotic transmission risk (8, 9).

Interferon (IFN) is crucial in protecting against coronavirus infection (10). Generally, it is believed that the canonical JAK-STAT signaling pathway mediates IFN to promote the transcription of IFN-stimulated genes (ISGs) (10, 11). Janus kinase 1 (JAK1) and tyrosine kinase 2 (TYK2) are essential mediators of the IFN response and have important immune system functions (12, 13). Signal transducer and activator of transcription 1 (STAT1) and STAT2 are the critical components that induce ISG production in the host antiviral response (14). CREB-binding protein (CBP) is an acetyltransferase acting on histones that promotes the acetylation of phosphorylated STAT1 (p-STAT1) (15, 16, 17). Furthermore, CBP forms a transcription enhancer with STAT1 and STAT2 to control ISG expression (18, 19).

After being released from cells, IFN attaches to the IFN alpha and beta receptor subunit 1 and IFN alpha and beta receptor subunit 2 to induce JAK1 and TYK2 phosphorylation (20, 21). Activated JAK1 and TYK2 stimulate STAT1 and STAT2 phosphorylation, respectively (22). Together with interferon regulatory factor-9 (IRF9), the activated STAT1 and STAT2 form the interferon-stimulated gene factor 3 (ISGF3) (18, 22). P-STAT1 and p-STAT2 expose the nuclear localization signal and associate with karyopherin α1 (23, 24). Then, the ISGF3 moves to the nucleus and binds the IFN-stimulated response elements (ISREs) to promote ISG production (25, 26). CBP induces p-STAT1 acetylation in the nucleus (27). Then, the acetylated STAT1 interacts with T cell protein tyrosine phosphatase (TCPTP) to trigger dephosphorylation, ultimately leading to STAT1 losing its DNA-binding activity and relocating to the cytoplasm (28). The STAT1 phosphorylation–acetylation–dephosphorylation cycle regulates JAK-STAT signaling pathway activation or silencing to maintain the balance of IFN signaling.

Cell cycle control is largely dependent on ubiquitination, which is an essential regulatory process (29, 30, 31). It is believed that ubiquitin (Ub) chain linkage differences result in different Ub protein functions (31). Among the eight potential homogeneous Ub chains, the canonical K48-linked Ub is critical in inducing the proteasomal degradation of cell cycle regulators (32). Furthermore, K11-linked and K29-linked Ub chains promote proteasomal degradation (33, 34). Additionally, only K63-linked Ub chains do not target proteins for proteasomal degradation (31).

Several studies provided evidence of the capacity of different coronaviruses to block IFN production and response by specifically targeting and degrading the host’s antiviral elements *via* the Ub-proteasome pathway. The severe actuate respiratory syndrome coronavirus 2 (SARS-CoV-2) ORF6 promotes the degradation of CHK1, a kinase involved in responding to DNA damage, through the proteasome pathway (35). SADS-CoV N protein interacts with RIG-I and induces its degradation through the Ub–proteasome pathway (36). No research to date has indicated the inhibition of IFN response by SADS-CoV nonstructural proteins through hijacking the Ub-proteasome system (UPS).

Previously, our study demonstrated that SADS-CoV nsp1 significantly inhibits IFN production (37). This inhibition was primarily attributed to nsp1 blocking of TBK1 Ub-like modification, which suppressed TBK1 phosphorylation. Furthermore, nsp1 induces CBP degradation through the Ub–proteasome pathway, thereby blocking IFN transcription enhancer formation. In this study, the potential of SADS-CoV nonstructural proteins to inhibit the IFN response was detected. SADS-CoV nsp1 was eventually identified as a potent antagonist in the IFN response. Nsp1 was important in IFN inhibition by SADS-CoV. This inhibition promoted SADS-CoV replication during the early stages of viral infection.

Despite earlier studies also demonstrated that coronavirus nsp1 blocks the JAK-STAT signaling pathway, the potential mechanism remains unclear. Our research indicated that SADS-CoV nsp1 inhibited the JAK-STAT signaling pathway by blocking STAT1 phosphorylation and acetylation. This blocking was attributed to the nsp1-induced JAK1 and CBP degradation. Overall, the investigation revealed new mechanisms by which SADS-CoV nsp1 blocked the IFN response. The findings contribute to comprehending the strategies utilized by coronavirus nsp1 to evade the host innate immune system.

## Results

### Nsp1 was crucial in SADS-CoV replication

**Figure 1:**
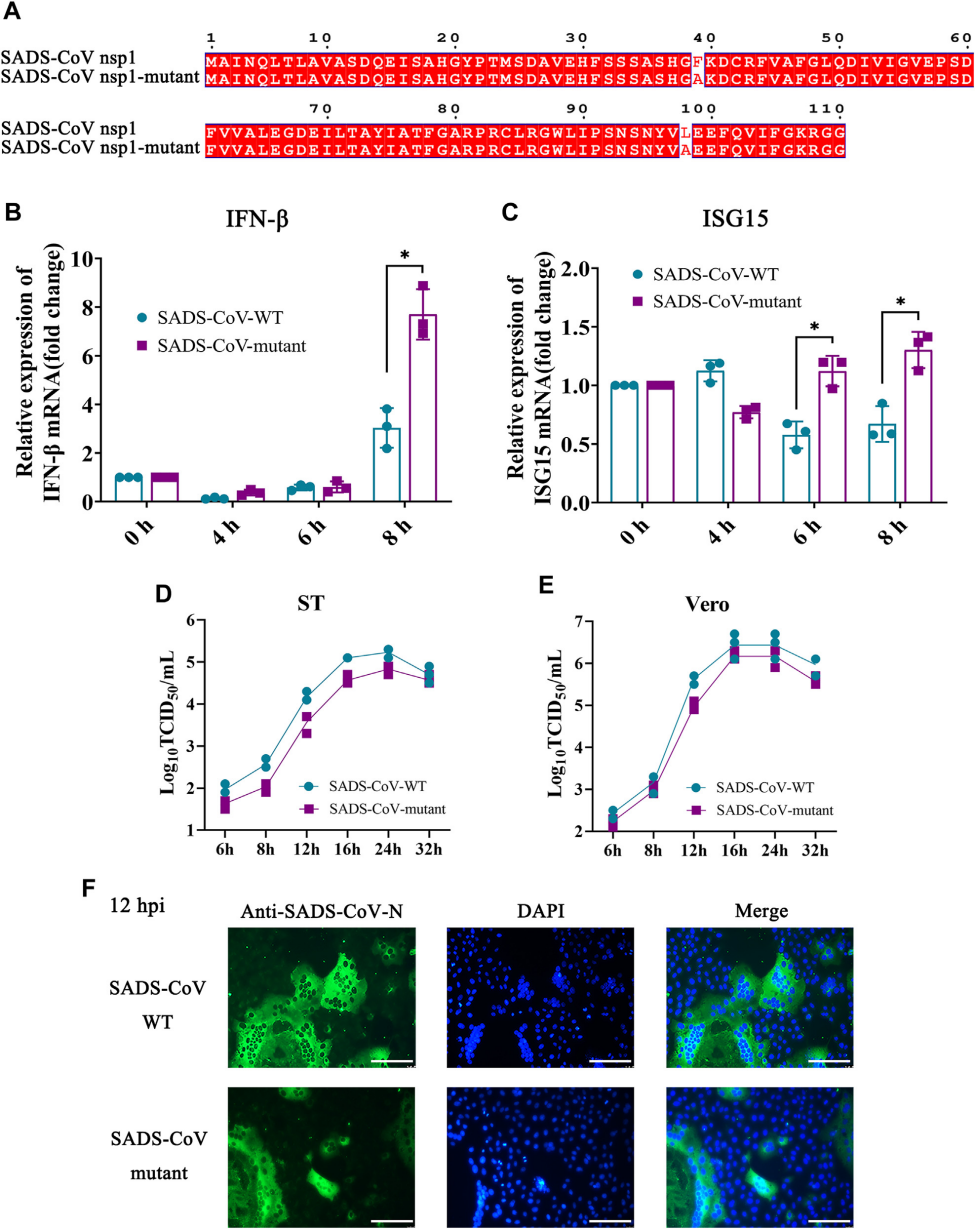
**Nsp1 was critical for virus replication.***A*, schematic diagram depicting the key amino acid sites in SADS-CoV nsp1. The alignment of nsp1 amino acids was conducted using the websites https://espript.ibcp.fr and https://www.genome.jp. *B* and *C*, LLC-PK1 cells were infected with SADS-CoV-WT or SADS-CoV-mutant at an MOI of 0.1. Subsequently, the cells were collected to detect the IFN-β and ISG15 mRNA levels using RT-qPCR. The data are the means ± SD. The *p*-value was calculated using the *t* test. ∗*p* < 0.05. *D* and *E*, Vero cells and ST cells were infected with SADS-CoV-WT or SADS-CoV-mutant at an MOI of 0.1. The infected cell supernatants were collected and titrated on Vero cells. The virus titers were determined by calculating the log_10_TCID_50_/ml. *F*, Vero cells were infected with SADS-CoV-WT or SADS-CoV-mutant at an MOI of 0.1 for 12 and 24 h. After 12 or 24 hpi, the cells were fixed, permeabilized, and blocked. The cells were then stained with SADS-CoV N mouse-pAb, followed by Alexa Fluor 488-conjugated goat anti-mouse staining (*green*). The nuclei were stained with DAPI (*blue*). Scale bar represents 200 μm. IFN-β, interferon-β; ISG, interferon-stimulated gene; MOI, multiplicity of infection; nsp1, nonstructure protein 1; SADS-CoV, swine acute diarrhea syndrome coronavirus.

Previously, the authors demonstrated that the Phe39 and Leu98 of SADS-CoV nsp1 were critical amino acids in antagonizing IFN production. These SADS-CoV nsp1 amino acids were mutated, ultimately generating a mutant virus (SADS-CoV-mutant) (Fig. 1*A*). Then, whether SADS-CoV-mutant infection inhibited IFN production and response was investigated. LLC-PK1 cells were infected with SADS-CoV-WT (wildtype) or SADS-CoV-mutant, and the cells were collected at 4, 6, and 8 h postinfection (hpi). The IFN-β and ISG15 mRNA levels were significantly increased in cells infected with SADS-CoV-mutant compared to the cells infected with SADS-CoV-WT. This result indicated that the SADS-CoV-mutant was not able to effectively inhibit IFN production and response (Fig. 1, *B* and *C*).

To determine if the ability of nsp1 to block the IFN system is essential for SADS-CoV replication, the SADS-CoV-WT and SADS-CoV-mutant growth kinetics were compared *via* a growth curve assay. In ST cells and Vero cells, the SADS-CoV-mutant produced lower quantities of progeny virions compared to SADS-CoV-WT from 8 hpi to 24 hpi. Due to the absence of IFN genes in Vero cells, this difference is more significant in ST cells than in Vero cells. (Fig. 1, *D* and *E*). The immunofluorescence assay result demonstrated that the proportion of cells infected with SADS-CoV-mutant was significantly reduced compared to cells infected with SADS-CoV-WT at 12 hpi (Fig. 1*F*). These results suggested that nsp1 was critical in SADS-CoV antagonism of IFN production and response. Additionally, the nsp1 mutations reduced the SADS-CoV replication capacity.

### Nsp1 blocked the IFN-β response pathway

**Figure 2:**
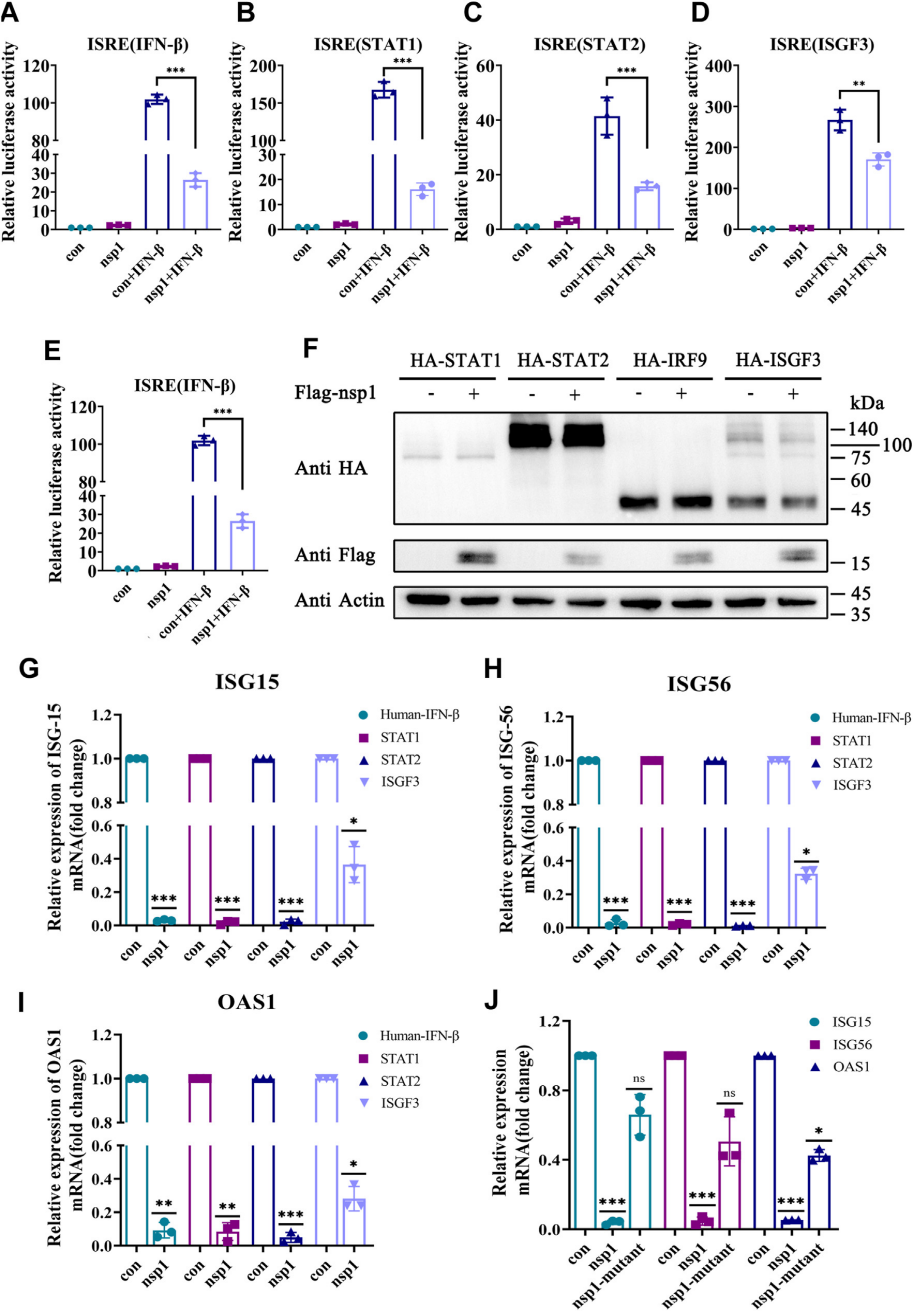
**Nsp1 inhibited the IFN response.***A–D*, HEK-293T cells were cotransfected with pCAGGS-2×HA-STAT1/pCAGGS-2×HA-STAT2/pCAGGS-2×HA-IRF9/ISGF3 (pCAGGS-2×HA-STAT1, pCAGGS-2×HA-STAT2, and pCAGGS-2×HA-IRF9), pIFN-β-luc, and pRL-TK at a ratio of 1:0.2:0.05. After 12 h, the cells were transfected with pCAGGS-3×Flag-nsp1. After 24 h, the cells were collected to detect ISRE promoter activity through the dual luciferase reporter assay. *E*, HEK-293T cells were co-transfected with pCAGGS-3×Flag-nsp1, pIFN-β-luc, and pRL-TK at a ratio of 1:0.2:0.05. After 24 h, the cells were stimulated by human IFN-β for 4 h. Then, the cells were collected to detect ISRE promoter activity through the dual luciferase reporter assay. *F*, HEK-293T cells were transfected with pCAGGS-2×HA-STAT1/pCAGGS-2×HA-STAT2/pCAGGS-2×HA-IRF9/ISGF3 (pCAGGS-2×HA-STAT1, pCAGGS-2×HA-STAT2, and pCAGGS-2×HA-IRF9). After 12 h, the cells were transfected with pCAGGS-3×Flag-nsp1. The cells were then collected to detect the nsp1, STAT1, STAT2, IRF9, and ISGF3 expression levels *via* Western blotting. *G–J*, HEK-293T cells were transfected with pCAGGS-2×HA-STAT1/pCAGGS-2×HA-STAT2/pCAGGS-2×HA-IRF9/ISGF3 (pCAGGS-2×HA-STAT1, pCAGGS-2×HA-STAT2, and pCAGGS-2×HA-IRF9). After 12 h, the cells were transfected with pCAGGS-3×Flag-nsp1. Then, the cells were collected to detect the OAS1, ISG15, and ISG56 mRNA levels using RT-qPCR. *J*, HEK-293T cells were transfected with pCAGGS-3×Flag-nsp1 or pCAGGS-3×Flag-nsp1-mutant. After 24 h, the cells were stimulated by SeV. Then, the cells were collected to detect the OAS1, ISG15, and ISG56 mRNA levels using RT-qPCR. Data are the mean ± SD. The *p*-value was calculated using the *t* test. ∗*p* < 0.05, ∗∗*p* < 0.01, ∗∗∗*p* < 0.001. HEK, human embryonic kidney cell line; IFN, interferon; IRF9, interferon regulatory factor-9; ISG, interferon-stimulated gene; ISGF3, interferon-stimulated gene factor 3; ISRE, interferon-stimulated response elements; nsp1, nonstructure protein 1; OAS1, 2′-5′-oligoadenylate synthetase 1; SeV, Sendai virus; STAT, signal transducer and activator of transcription.

STAT1 and STAT2 combine to form ISGF3 with IRF9 and subsequently induce ISRE-driven ISG transcription in the nucleus. Whether nsp1 inhibited the ISRE promoter activity stimulated by STAT1, STAT2, IRF9, or ISGF3 was detected to explore the potential regulatory role of SADS-CoV nsp1 in the IFN response. The ISRE promoter activity was examined using a dual-luciferase reporter assay, whereas the exogenous protein expression was detected *via* Western blotting. Compared to the negative control, STAT1, STAT2, IRF9, ISGF3, and human IFN-β significantly induced ISRE promoter activation. Contrastingly, nsp1 significantly inhibited ISRE promoter activity in cells stimulated by human IFN-β, STAT1, STAT2, or IRF9 (Fig. 2, *A*–*C* and *E*). However, this inhibition was attenuated in the ISFG3-stimulated cells, suggesting that nsp1 blocked the IFN response by targeting ISGF3 complex formation (Fig. 2*D*). Furthermore, nsp1 had no negative effect on exogenous STAT1, STAT2, IRF9, and ISGF3 expression levels (Fig. 2*F*).

The suppressive effect of nsp1 on ISG15, ISG56, and 2′-5′-oligoadenylate synthetase 1 (OAS1) mRNA levels was investigated. Real-time PCR analyses revealed that nsp1 efficiently inhibited ISG15, ISG56, and OAS1 expression in cells stimulated by human IFN-β, STAT1, STAT2, or IRF9 (Fig. 2, *G*–*I*). Furthermore, Phe39 and Lys98 in the nsp1 sequence were mutated to alanine, generating a nsp1-mutant. Compared with the nsp1-transfected cells, the ISG15, ISG56, and OAS1 mRNA levels were not significantly inhibited in the cells transfected with the nsp1-mutant. This result suggested that Phe39 and Lys98 mutation might lead to the loss of nsp1 function in antagonizing the IFN response (Fig. 2*J*). These findings indicated that SADS-CoV nsp1 strongly inhibited the IFN response.

### Nsp1 inhibited STAT1 phosphorylation

**Figure 3:**
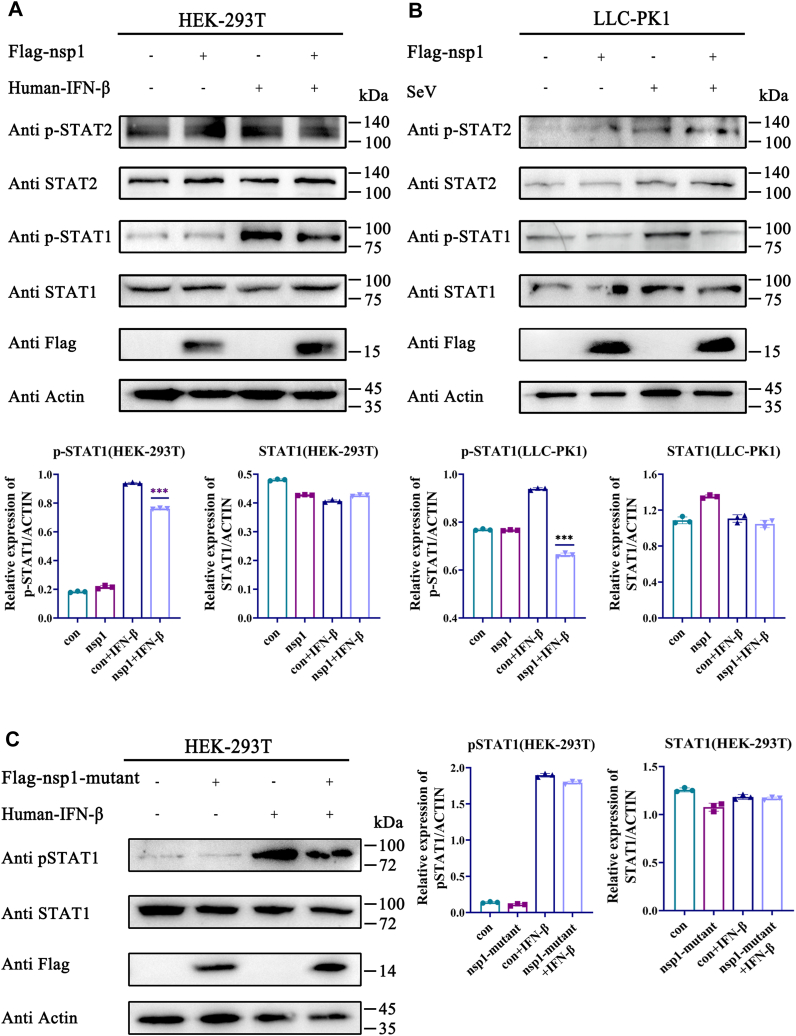
**Nsp1 inhibited STAT1 phosphorylation.***A* and *B*, HEK-293T cells and LLC-PK1 cells were transfected with pCAGGS-3×Flag-nsp1. After 24 h, HEK-293T cells were incubated with human IFN-β for 4 h, and LLC-PK1 cells were stimulated by SeV for 8 h. STAT1, p-STAT1, STAT2, and p-STAT2 expression was detected by Western blotting. All protein levels were analyzed using ImageJ. Western blotting assay was repeated in two independent experiments. *C*, HEK-293T cells were transfected with pCAGGS-3×Flag-nsp1-mutant. After 24 h, HEK-293T cells were incubated with human IFN-β for 4 h. STAT1 and p-STAT1 expression was detected by Western blotting. All protein levels were analyzed using ImageJ. Western blotting assay was repeated in two independent experiments. The data are the means ± SD. The *p*-value was calculated using the *t* test. ∗∗∗*p* < 0.001. HEK, human embryonic kidney cell line; IFN-β, interferon-β; nsp1, nonstructure protein 1; SeV, Sendai virus; STAT, signal transducer and activator of transcription.

STAT1 and STAT2 are crucial transcription activators in the JAK–STAT pathway. Endogenous STAT1 and STAT2 expression and phosphorylation were detected by Western blotting to investigate the effect of nsp1 and nsp1-mutant on these proteins. The result demonstrated that nsp1 decreased STAT1 phosphorylation in human embryonic kidney cell line (HEK)-293T cells stimulated by human IFN-β and LLC-PK1 cells induced by Sendai virus (SeV). The nsp1-mutant did not show a similar function (Fig. 3, *A*–*C*). Nevertheless, nsp1 did not block STAT2 phosphorylation. Nsp1 expression did not influence STAT1 and STAT2 endogenous expression, suggesting that nsp1 specifically inhibited STAT1 phosphorylation (Fig. 3, *A* and *C*). Furthermore, nsp1 induced IRF9 degradation (data not shown). These results indicated that STAT1 and IRF9 are the primary targets of nsp1-mediated inhibition of the IFN response.

### Nsp1 degraded JAK1

**Figure 4:**
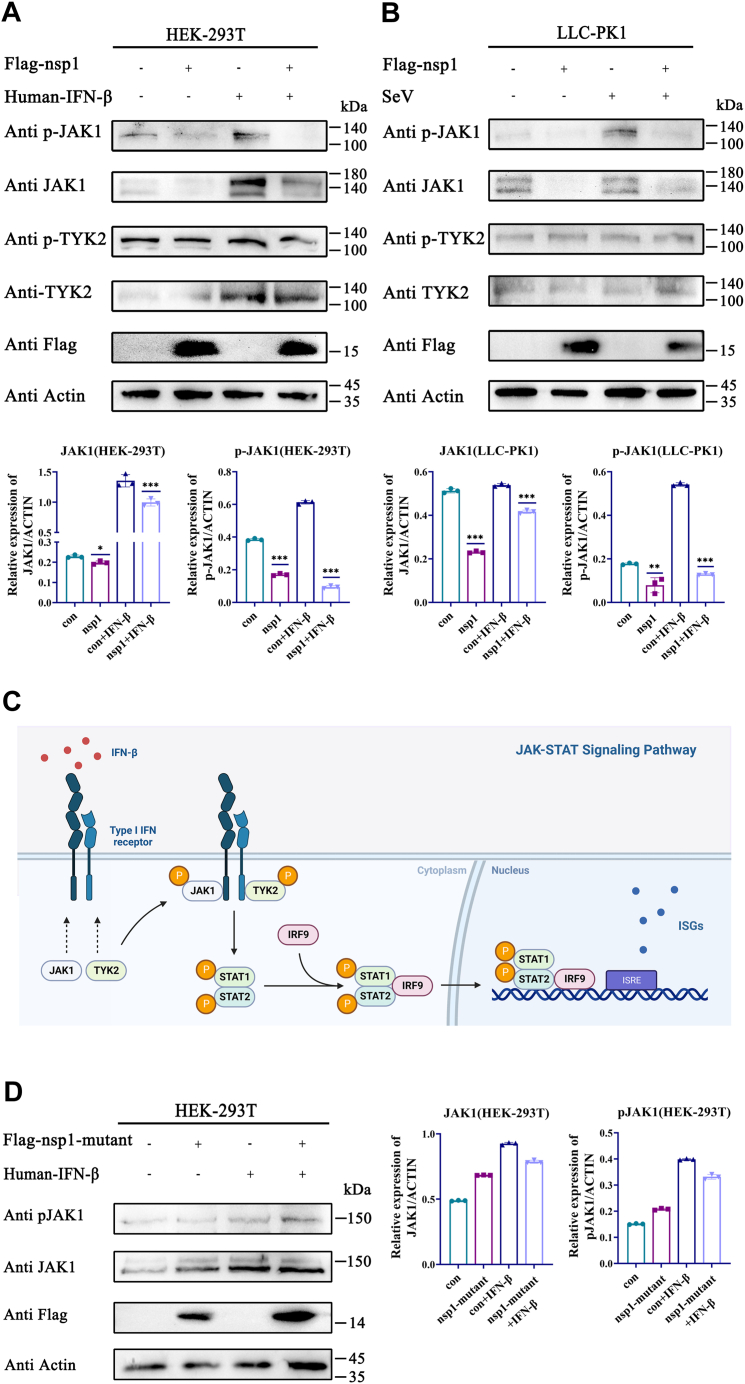
**Nsp1 induced JAK1 degradation.***A* and *B*, HEK-293T cells and LLC-PK1 cells were transfected with pCAGGS-3×Flag-nsp1. After 24 h, the HEK-293T cells were incubated with human IFN-β for 4 h, and the LLC-PK1 cells were stimulated by SeV for 8 h. JAK1, p-JAK1, TYK2, and p-TYK2 expression was detected by Western blotting. All protein levels were analyzed using ImageJ. Western blotting assay was repeated in two independent experiments. *C*, schematic presentation of the ISGF3 activation process created using BioRender.com. *D*, HEK-293T cells were transfected with pCAGGS-3×Flag-nsp1-mutant. After 24 h, HEK-293T cells were incubated with human IFN-β for 4 h. JAK1 and p-JAK1 expression was detected by Western blotting. All protein levels were analyzed using ImageJ. Western blotting assay was repeated in two independent experiments. The data are the means ± SD. The *p*-value was calculated using the *t* test. ∗*p* < 0.05, ∗∗*p* < 0.01, and ∗∗∗*p* < 0.001. HEK, human embryonic kidney cell line; IFN-β, interferon-β; ISGF3, interferon-stimulated gene factor 3; JAK1, Janus kinase 1; nsp1, nonstructure protein 1; SeV, Sendai virus; TYK2, tyrosine kinase 2.

After observing the nsp1-induced inhibition of STAT1 phosphorylation, the investigation was subsequently focused on the endogenous expression and phosphorylation of JAK1 and TYK2, which are crucial kinases for inducing STAT1 activation. Nsp1 efficiently induced JAK1 degradation in HEK-293 cells and LLC-PK1 cells, whereas the nsp1-mutant did not induce the JAK1 degradation (Fig. 4, *A*–*D*). Contrastingly, endogenous TYK2 expression and phosphorylation were not inhibited in the nsp1-expressing cells (Fig. 4, *A* and *B*). The results demonstrated that nsp1 might degrade JAK1 to inhibit STAT1 phosphorylation, thereby blocking the JAK-STAT signaling pathway.

### Nsp1 degraded JAK1 *via* the proteasome pathway

**Figure 5:**
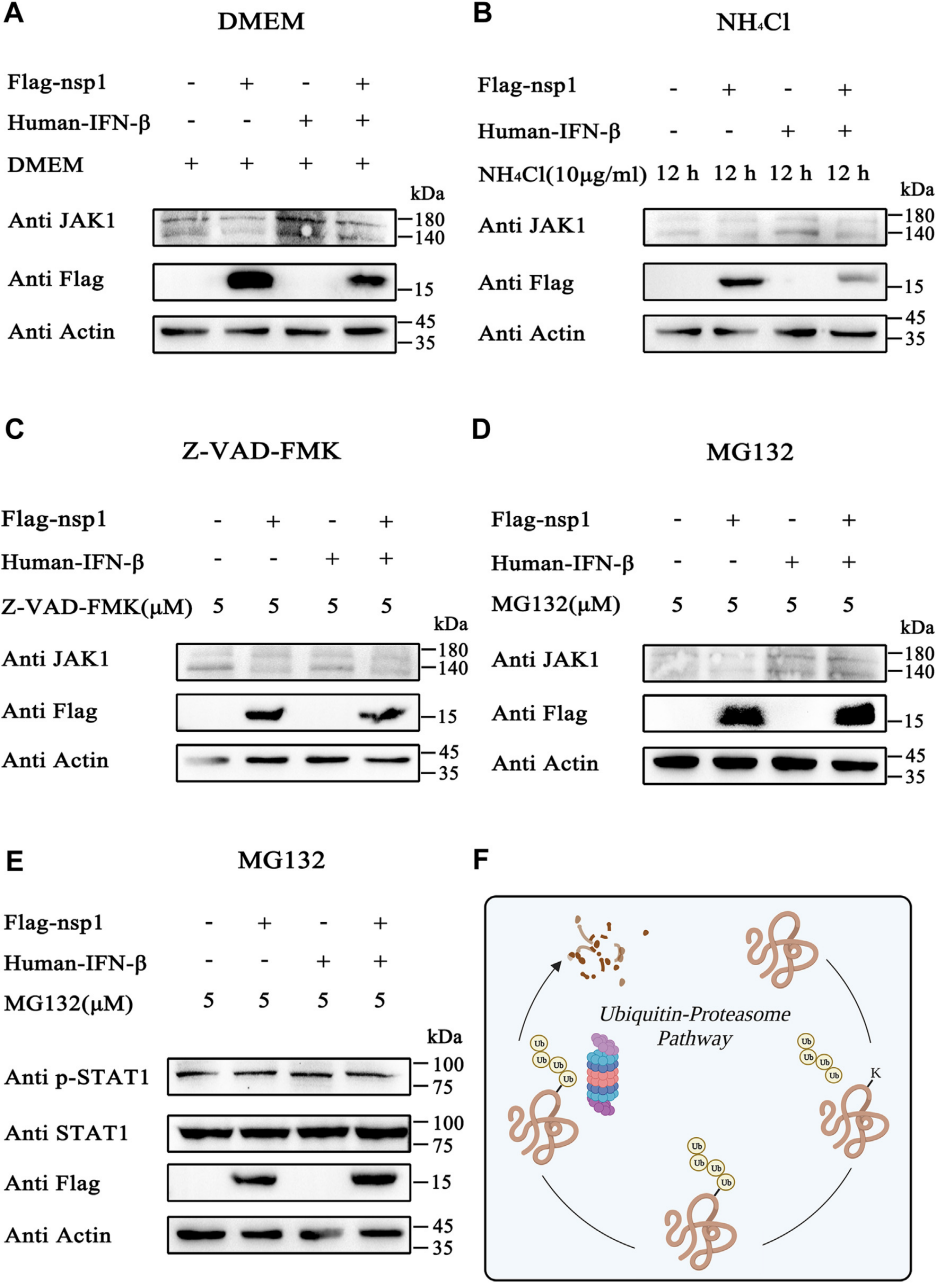
**Nsp1 degraded JAK1 through the proteasome pathway*.****A–E*, HEK-293T cells were transfected with pCAGGS-3×Flag-nsp1. After 24 h, DMEM, MG132, Z-VAD-FMK, and NH_4_Cl were added. *A*, cells were incubated with DMEM for 6 h. *B*, NH_4_Cl (working concentration 10 μg/ml) was added to the cells, and the cells were collected after 12 h. *C*, Z-VAD-FMK (working concentration 5 μM) was added to cells, and the cells were collected after 6 h. *D* and *E*, MG132 (working concentration 5 μM) was added to the cells, and the cells were collected after 6 h. *A–E*, JAK1, STAT1, and p-STAT1 expression levels were analyzed by Western blotting. *F*, schematic presentation of the ubiquitin-proteasome pathway created using BioRender.com. DMEM, Dulbecco’s modified Eagle’s medium; HEK, human embryonic kidney cell line; IFN-β, interferon-β; JAK1, Janus kinase 1; nsp1, nonstructure protein 1; STAT, signal transducer and activator of transcription.

The pathway of JAK1 degradation by nsp1 was analyzed to investigate the mechanism through which nsp1 downregulated JAK1 expression. Specifically, HEK-293T cells were treated with Dulbecco’s modified Eagle’s medium (DMEM, the negative control), 10 μM MG132 (a proteasome pathway inhibitor), 5 μM Z-VAD-FMK (an apoptosis pathway inhibitor), or 10 μg/ml NH_4_Cl (an autophagy pathway inhibitor). Nsp1 persistently induced the degradation of endogenous JAK1 in all treated cells (Fig. 5, *A*–*C*). However, this degradation was effectively attenuated in cells treated with 5 μM MG132, suggesting that MG132 is an effective antagonist against nsp1-induced JAK1 degradation (Fig. 5*D*). These results indicated that nsp1 degraded JAK1 through the proteasome pathway.

Subsequently, it was detected that nsp1 inhibited the STAT1 phosphorylation level in MG132-treated cells. Compared to the negative control, nsp1 did not induce JAK1 degradation and inhibit STAT1 phosphorylation (Fig. 5*E*). Overall, the results indicated that nsp1 inhibited STAT1 phosphorylation by inducing JAK1 degradation *via* the proteasome pathway.

### Nsp1 promoted K11/K48-linked JAK1 polyubiquitination

**Figure 6:**
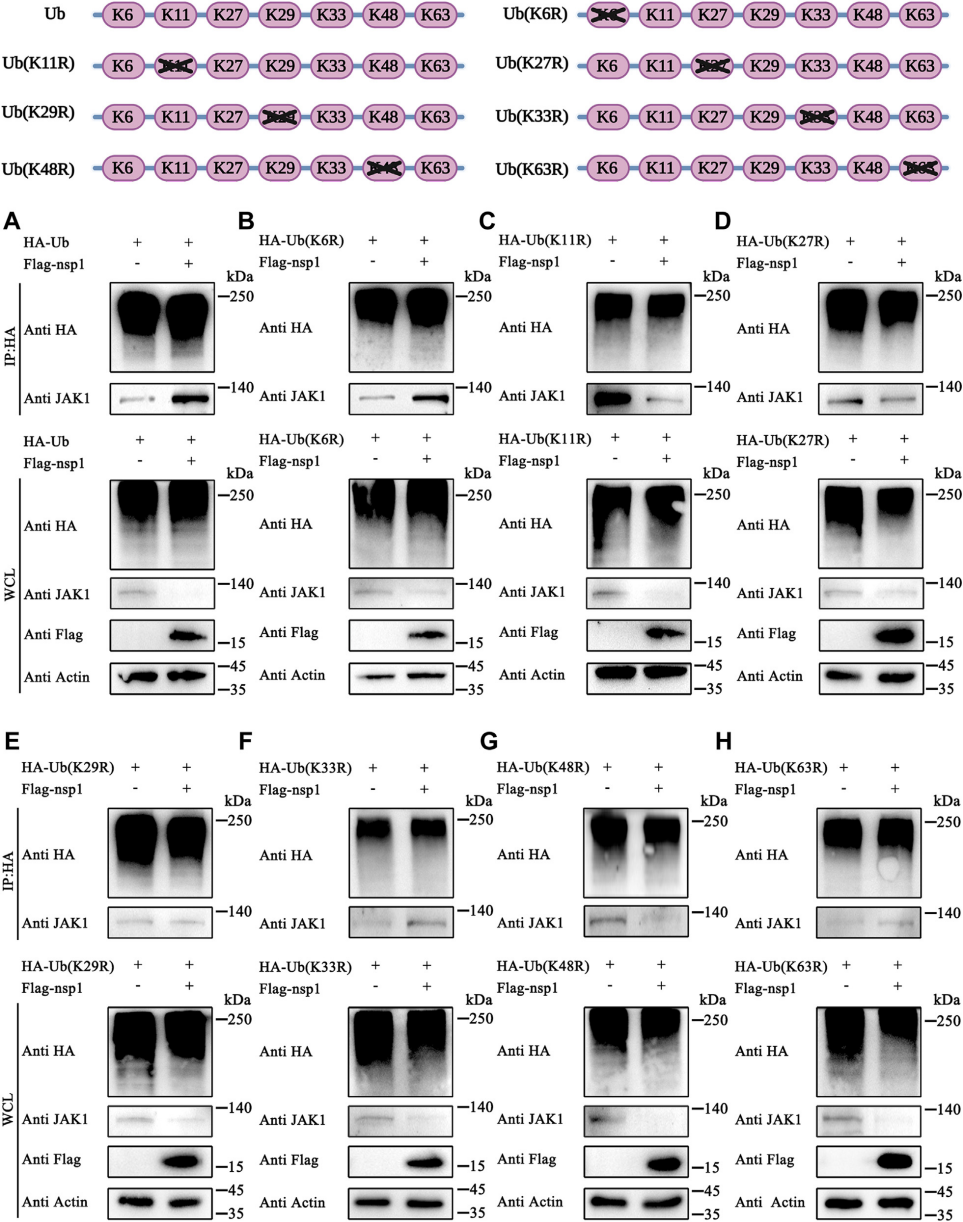
**Nsp1 induced K11/K48 linked JAK1 ubiquitination.***A–H*, HEK-293T cells were transfected with pCAGGS-2×HA-Ub or plasmids expressing Ub mutants (Ub-K6R, Ub-K11R, Ub-27R, Ub-K29R, Ub-K33R, Ub-K48R, and Ub-K63R) at a concentration of 2500 ng per well in 6-well plates. After 12 h, the cells were transfected with pCAGGS-3×Flag-nsp1 (1000 ng per well). After 24 h, the cells were collected and incubated with HA-tagged beads. Subsequently, the interaction between Ub (or Ub mutants) and JAK1 was detected using Western blotting. HEK, human embryonic kidney cell line; JAK1, Janus kinase 1; nsp1, nonstructure protein 1; Ub, ubiquitin.

Protein degradation mediated by the Ub-proteasome pathway can occur *via* monoubiquitination or polyubiquitination. HEK-293T cells were transfected with Ub or Ub mutants (Ub-K6R, Ub-K11R, Ub-27R, Ub-K29R, Ub-K33R, Ub-K48R, and Ub-K63R) to investigate the specific form of nsp1-induced JAK1 ubiquitination. The Ub mutants were characterized by replacing the lysine residues (K6, K11, K27, K29, K33, K48, or K63) with arginine. After 12 h, the cells were transfected with pCAGGS-3×Flag-nsp1. Then, the cells were collected to detect the interaction between JAK1 and Ub (or Ub mutants) by Western blotting. Nsp1 induced the interaction between JAK1 and Ub (Fig. 6*A*). Furthermore, nsp1 promoted interaction between JAK1 and Ub-K6R, Ub-K27R, Ub-K29R, and Ub-K63R, indicating that silencing K6, K27R, K29, and K63 of Ub did not significantly impact JAK1 degradation (Fig. 6, *B*, *D–F* and *H*). Conversely, nsp1 did not induce interaction between JAK1 and Ub-K11R and Ub-K48R, suggesting that K11 and K48 were critical lysines in nsp1-induced JAK1 degradation (Fig. 6, *C* and *G*).

**Figure 7:**
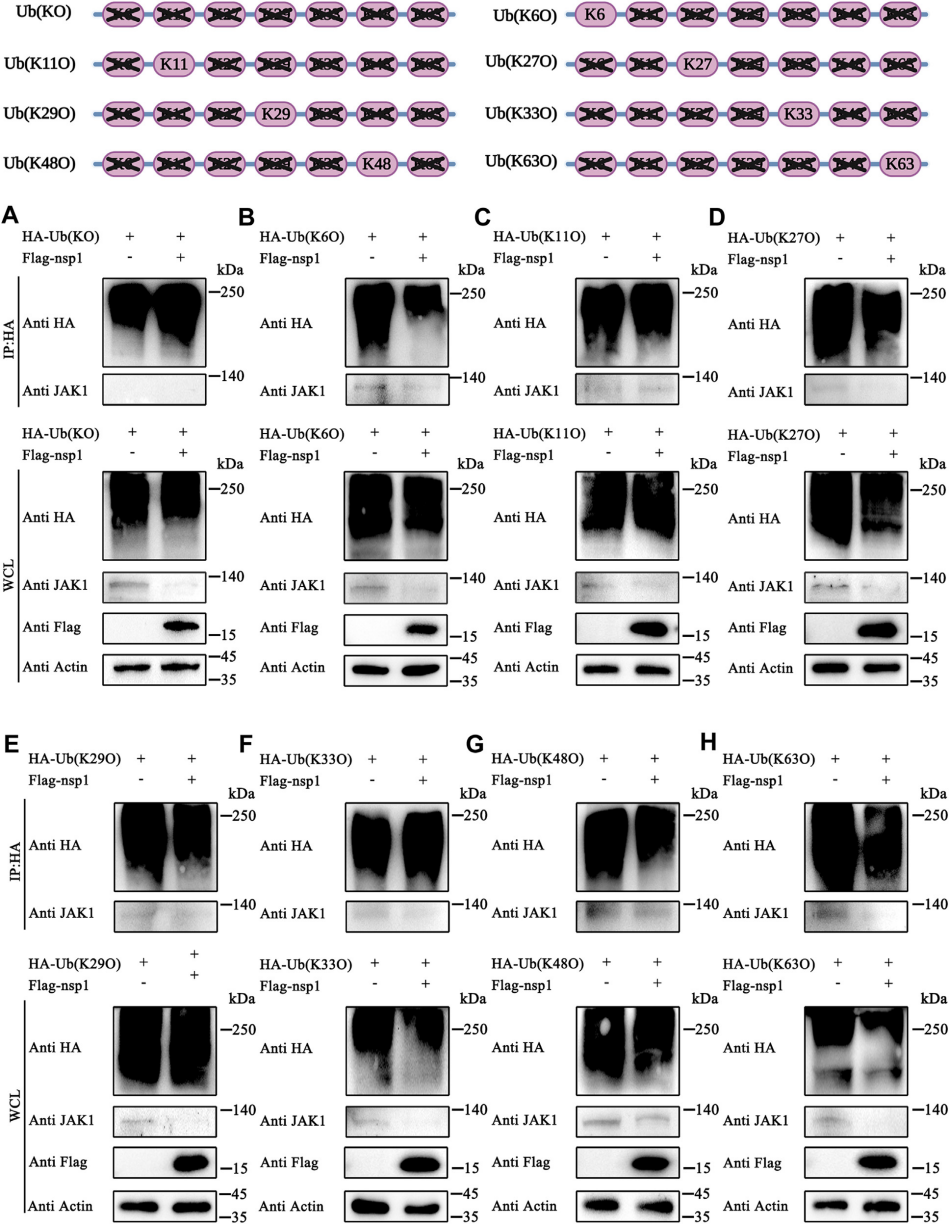
**Nsp1 induced K11/K48 linked JAK1 ubiquitination.***A–H*, HEK-293T cells were transfected with plasmids expressing Ub mutants (Ub-KO, Ub-K6O, Ub-K11O, Ub-27O, Ub-K29O, Ub-K33O, Ub-K48O, and Ub-K63O) at a concentration of 2500 ng per well in 6-well plates. After 12 h, the cells were then transfected with pCAGGS-3×Flag-nsp1 (1000 ng per well). After 24 h, the cells were collected and incubated with HA-tagged beads. Subsequently, the interaction between Ub mutants and JAK1 was detected using Western blotting. HEK, human embryonic kidney cell line; JAK1, Janus kinase 1; nsp1, nonstructure protein 1; Ub, ubiquitin.

The above results were validated by detecting the interaction between JAK1 and other Ub mutants, including Ub-KO (a mutated type of Ub where all seven lysine residues are replaced with arginine), Ub-K6O (a mutated type of Ub where all lysine residues except lysine 6 are replaced with arginine), Ub-K11O, Ub-K27O, Ub-K29O, Ub-K33O, Ub-K48O, and Ub-K63O. Nsp1 induced JAK1 ubiquitination in the cells expressing Ub-K11O and Ub-K48O (Fig. 7, *C* and *G*). Additionally, nsp1 did not induce the interaction between JAK1 and Ub-KO, Ub-K6O, Ub-K27O, Ub-K29O, and Ub-K63O (Fig. 7, *A*, *B*, *D–F* and *H*). These results indicated that nsp1 degraded JAK1 through K11 and K48-linked polyubiquitination.

### Nsp1 blocked p-STAT1 acetylation and dephosphorylation

**Figure 8:**
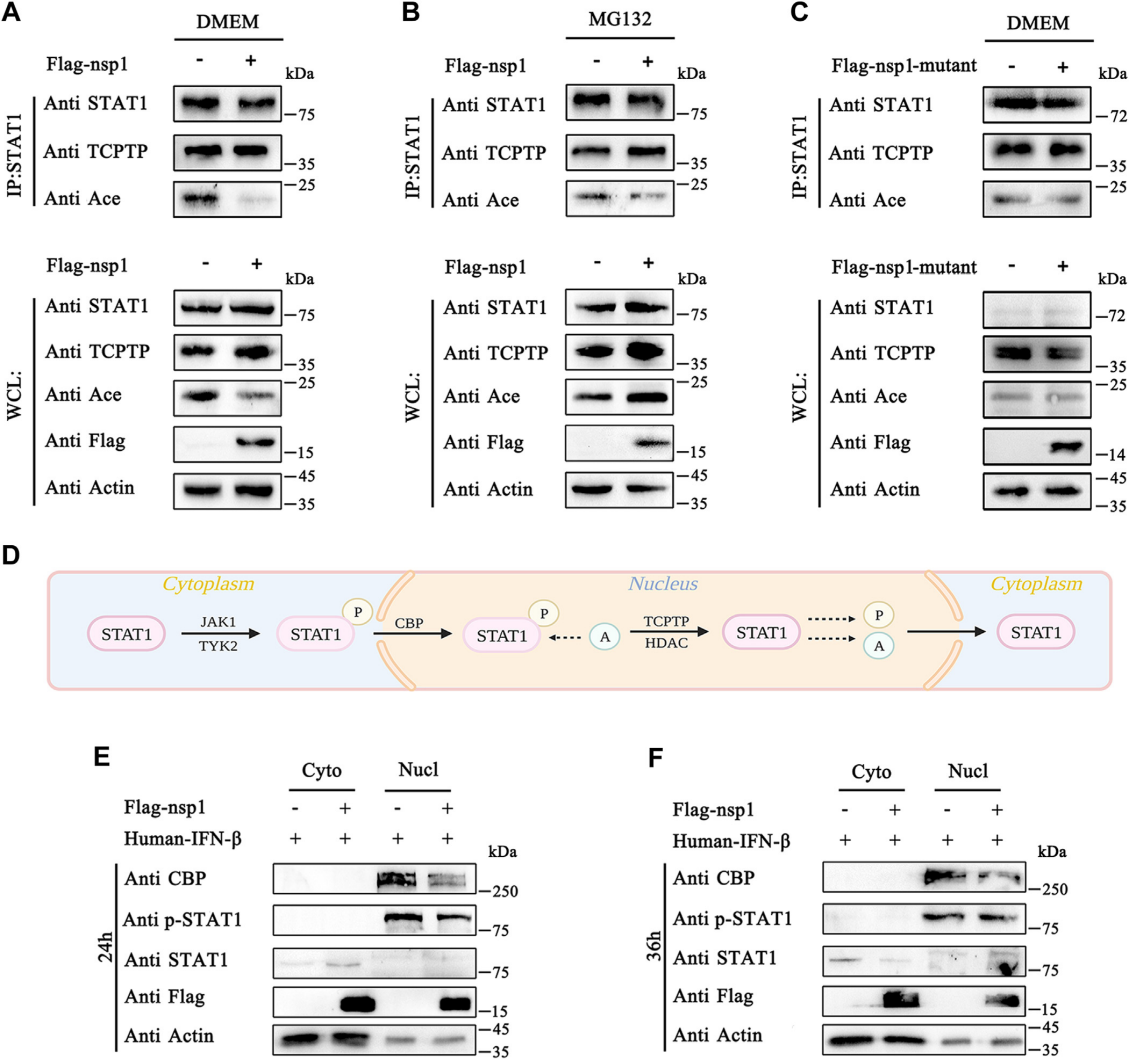
**Nsp1 inhibited STAT1 acetylation and dephosphorylation.***A* and *B*, HEK-293T cells were plated onto 6-well plates and transfected with pCAGGS-3×Flag-nsp1. Then, the cells were incubated with DMEM or MG132 (5 μM) for 6 h. The cells were collected and incubated with STAT1-tagged beads. The interaction between STAT1 and TCPTP/Ace was detected using Western blotting. *C*, HEK-293T cells were plated onto 6-well plates and transfected with pCAGGS-3×Flag-nsp1-mutant. The cells were incubated with DMEM for 6 h. Then, the cells were collected and incubated with STAT1-tagged beads. The interaction between STAT1 and TCPTP/Ace was detected using Western blotting. *D*, schematic presentation of the STAT1 phosphorylation-acetylation-dephosphorylation cycle created using BioRender.com. *E* and *F*, HEK-293T cells were plated in 60-mm glass-bottom dishes and transfected with pCAGGS-3×Flag-nsp1 (5000 ng per dish). The cells were collected after 24 or 36 h. Nuclear and cytoplasmic proteins were extracted using a protein extraction kit. Then, the CBP and STAT1 expression levels and STAT1 phosphorylation level were detected by Western blotting. CBP, CREB-binding protein; DMEM, Dulbecco’s modified Eagle’s medium; HEK, human embryonic kidney cell line; IFN-β, interferon-β; JAK1, Janus kinase 1; nsp1, nonstructure protein 1; STAT, signal transducer and activator of transcription; TCPTP, T cell protein tyrosine phosphatase; TYK2, tyrosine kinase 2.

In the nucleus, p-STAT1 interacts with CBP to induce ISG transcription and undergoes acetylation. The acetylated STAT1 binds TCPTP to undergo dephosphorylation. HEK-293T cells were transfected with pCAGGS-3×Flag-nsp1 and stimulated by human IFN-β to detect whether nsp1 inhibited STAT1 acetylation and dephosphorylation. The cells were collected to detect STAT1 acetylation and the interaction between STAT1 and TCPTP. Nsp1 significantly inhibited STAT1 acetylation (Fig. 8*A*). Based on previous findings on nsp1-induced CBP degradation, it was concluded that nsp1 inhibited STAT1 acetylation by degrading CBP. Furthermore, nsp1 did not affect TCPTP expression. However, it slightly inhibited the interaction between STAT1 and TCPTP, suggesting that nsp1 blocked STAT1 dephosphorylation by inhibiting STAT1 acetylation (Fig. 8*A*). To verify these results, the cells were treated with MG132 to eliminate the nsp1 function in degrading CBP, and the STAT1 acetylation and dephosphorylation were detected. The nsp1 inhibition of STAT1 acetylation and dephosphorylation was significantly attenuated when CBP was no longer degraded (Fig. 8*B*). Additionally, nsp1-mutant did not inhibit STAT1 acetylation and dephosphorylation (Fig. 8*C*).

Nsp1 inhibition of STAT1 acetylation might block STAT1 export from the nucleus to the cytoplasm. HEK-293T cells were transfected with pCAGGS-3×Flag-nsp1 to verify this finding. At 24 h or 36 h post-transfection, the cells underwent nucleocytoplasmic fractionation. Nsp1 significantly degraded CBP in both the 24-h and 36-h groups (Fig. 8, *D* and *E*). In the 24-h group, nsp1 increased STAT1 content in the cytoplasm and decreased p-STAT1 content in the nucleus by inhibiting STAT1 phosphorylation. In the 36-h group, STAT1 content in the cytoplasm was decreased, and p-STAT1 content in the nucleus was increased, which was due to nsp1 blocking STAT1 acetylation. These results suggested that nsp1 blocked STAT1 nuclear translocation by inhibiting STAT1 phosphorylation and prevented STAT1 translocation out of the nucleus by inhibiting STAT1 acetylation.

## Discussion

Host cells adopt multiple strategies to defend against coronaviruses, including the IFN response. Simultaneously, coronaviruses have developed various methods to evade the host’s innate immune response by blocking the IFN response. Increasing evidence indicates that coronavirus nonstructural proteins are important in suppressing the IFN response by targeting the JAK-STAT signaling pathway. For example, porcine deltacoronavirus nsp5 cleaves STAT2 to block ISGF3 formation (38). SARS-CoV-2 nsp13 and ORF6 block STAT1 nuclear translocation to inhibit ISG transcription (39). However, the mechanism of SADS-CoV nonstructural proteins in inhibiting the IFN response remains unknown. In this study, SADS-CoV nonstructural proteins that inhibit the IFN response were identified. The results demonstrated that SADS-CoV nsp1 was the most potent antagonistic influence on the IFN response by blocking the JAK-STAT signaling pathway. Additionally, the study yielded novel findings that demonstrated the mechanisms of SADS-CoV nsp1 in IFN response inhibition, which provided valuable insights into SADS-CoV evasion of the host’s innate immune response.

It is generally believed that coronavirus nsp1 induces host mRNA degradation (40, 41, 42). Coronavirus nsp1 also inhibits host protein translation by interacting with the 40S ribosome subunit, ultimately inhibiting the IFN system (41, 42). However, accumulating evidence suggests that coronavirus nsp1 also inhibits the IFN system, which is separate from its ability to inhibit host protein translation and mRNA degradation (43, 44, 45, 46, 47, 48, 49, 50). In the present study, mutation of SADS-CoV nsp1 critical amino acids (Phe39 and Lys98) significantly diminished its ability to inhibit the IFN response. Consequently, the nsp1 mutations reduced the replicative capacity of SADS-CoV during the early stages of viral infection. Further research demonstrated that SADS-CoV nsp1 significantly inhibited ISRE promoter activity and ISG mRNA levels, suggesting that SADS-CoV nsp1 was a potent antagonist in blocking the IFN response induced by antiviral molecules (STAT1, STAT2, and IRF9). These results indicated that SADS-CoV nsp1 antagonized the IFN response by targeting the ISGF3 complex, which acts as an important virulence factor for SADS-CoV.

**Figure 9:**
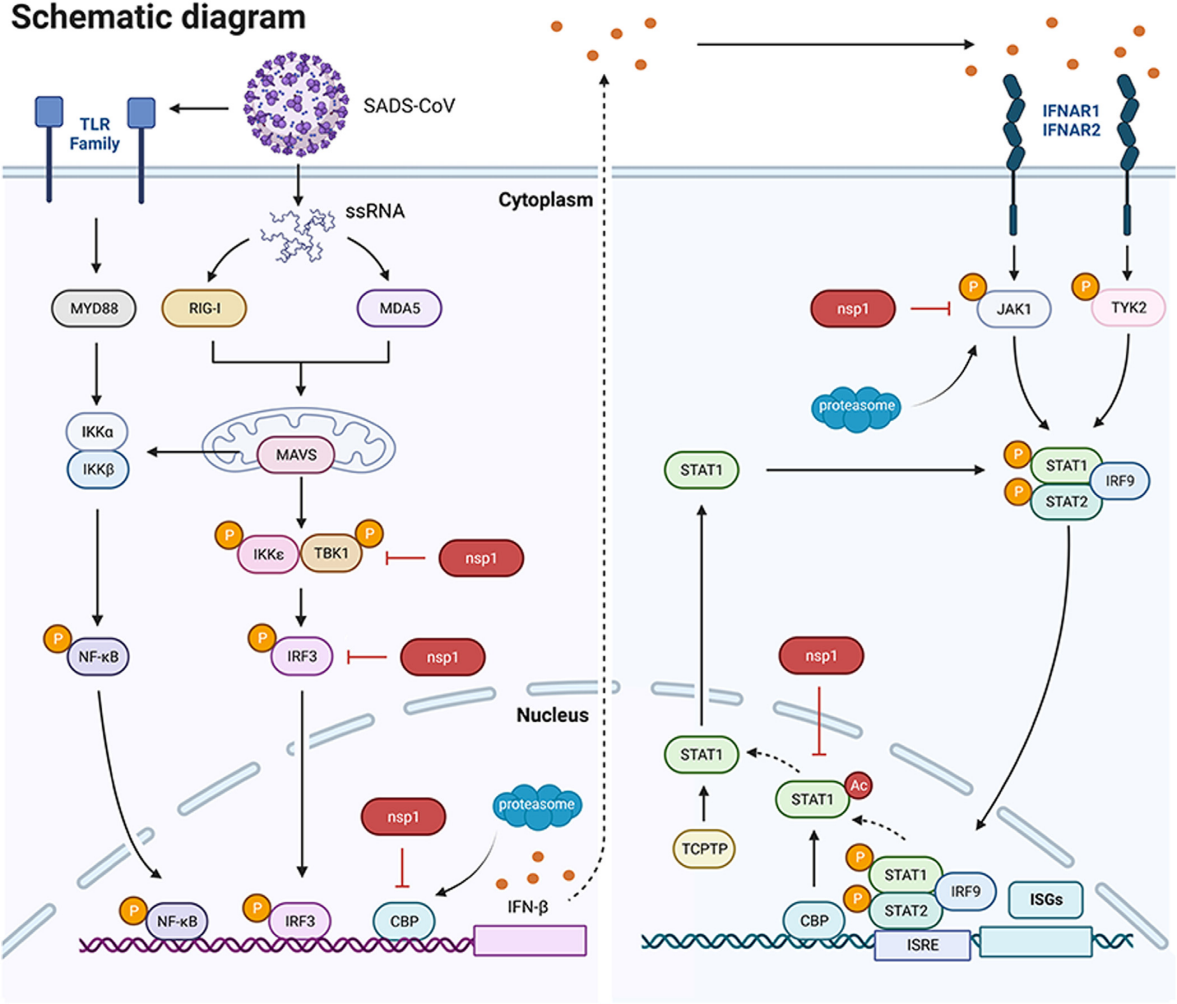
**Schematic diagram of SADS-CoV nsp1 blocking IFN production and response.** SADS-CoV nsp1 inhibited TBK1 phosphorylation by preventing TBK1 ubiquitin modification, ultimately blocking IRF3 activation. SADS-CoV nsp1 blocked IFN transcriptional enhancer formation by inducing CBP degradation. SADS-CoV nsp1 promoted K11/K48-linked JAK1 polyubiquitination, then induced JAK1 degradation through the proteasome pathway. SADS-CoV inhibited STAT1 phosphorylation by inducing JAK1 degradation. SADS-CoV nsp1 inhibited STAT1 acetylation and dephosphorylation by inducing CBP degradation. This schematic diagram was created using BioRender.com. CBP, CREB-binding protein; IFN-β, interferon-β; IFNAR, interferon alpha and beta receptor subunit; IRF9, interferon regulatory factor-9; ISG, interferon-stimulated gene; ISGF3, interferon-stimulated gene factor 3; ISRE, interferon-stimulated response elements; JAK1, Janus kinase 1; nsp1, nonstructure protein 1; SADS-CoV, Swine Acute Diarrhea Syndrome Coronavirus; STAT, signal transducer and activator of transcription; TCPTP, T cell protein tyrosine phosphatase; TYK2, tyrosine kinase 2.

Coronavirus nsp1 significantly blocks the IFN system by inhibiting the expression or phosphorylation of antiviral proteins. For α-coronavirus, transmissible gastroenteritis virus nsp1 inhibited IFN-β promoter activities. However, the specific molecular target(s) remain unknown (43). Porcine epidemic diarrhea virus (PEDV) nsp1 is the most potent IFN antagonist among all PEDV proteins, which inhibits STAT1 phosphorylation and degrades CBP (44, 45, 46). Furthermore, PEDV nsp1 does not affect IRF3 phosphorylation and nuclear localization (45). For β-coronavirus, SARS-CoV-1 nsp1 degrades JAK1 and inhibits IRF3, IRF7, and STAT1 phosphorylation (47). SARS-CoV-2 nsp1 inhibits IFN production by suppressing multiple IRF3 signal pathway targets and blocks the IFN response by inhibiting STAT1 phosphorylation (48, 49, 50). Thus, coronavirus nsp1 targets multiple antiviral proteins and displays a potent ability to suppress the host’s innate immune response. Previously, our study demonstrated that SADS-CoV nsp1 significantly inhibited IFN production by suppressing IRF3 phosphorylation and inducing CBP degradation (37). In the present study, it was determined that SADS-CoV nsp1 blocked the IFN response by inhibiting STAT1 phosphorylation and acetylation (Fig. 9). These results suggested that SADS-CoV nsp1 also targets multiple antiviral proteins, which potently blocks the IFN system to create a cellular environment favorable for SADS-CoV replication.

IFN signal transduction relies on the normal phosphorylation and nuclear translocation of STAT1 (50, 51). STAT1 can form homodimers that mediate a noncanonical JAK-STAT signaling pathway (STAT1/STAT1-driven), while STAT2 does not have a similar function (52). These studies indicated that STAT1 is dominant in the JAK-STAT signaling pathway. Thus, coronavirus nsp1 inhibition of STAT1 phosphorylation instead of STAT2 was more effective in suppressing the IFN response. Although STAT1 is considered an important target for coronavirus nsp1, it remains unclear how SADS-CoV nsp1 inhibits STAT1 phosphorylation. In this study, it was determined that SADS-CoV nsp1 induced JAK1 degradation to inhibit STAT1 phosphorylation. Further investigation demonstrated that nsp1 promoted K11- and K48-linked JAK1 polyubiquitination, resulting in JAK1 degradation through the Ub–proteasome pathway.

Prolonged activation of the IFN response can damage the host’s innate immune system (53). The balance between STAT1 phosphorylation and dephosphorylation is important for the IFN response (54). STAT1 activation is rapid but transient to maintain the normal IFN response. JAK1 induces STAT1 activation, while CBP and TCPTP negatively regulate STAT1 activity by promoting STAT1 acetylation and dephosphorylation (27, 28). Multiple coronavirus proteins block the IFN response by inhibiting STAT1 phosphorylation and nuclear translocation. However, it has not been determined that coronavirus proteins inhibit the IFN response by targeting STAT1 acetylation and phosphorylation. In the present study, SADS-CoV nsp1 inhibited the CBP-mediated STAT1 acetylation by inducing CBP degradation, then blocked the interaction between TCPTP and STAT1 to suppress STAT1 dephosphorylation. This effect inhibited STAT1 transport to the cytoplasm and prevented STAT1 from receiving antiviral signaling through JAK1 and TYK2, delaying the IFN response. Although sustaining nuclear STAT1 levels is critical for the IFN response, the CBP degradation induced by SADS-CoV nsp1 inhibited STAT1 formation of the STAT1–CBP complex and prevented ISG production. For SADS-CoV, it would be a more efficient and powerful immune evasion strategy that inhibits the upstream process (STAT1 phosphorylation) and downstream process (STAT1 acetylation and dephosphorylation) of the JAK-STAT signaling pathway.

Much research has demonstrated that regulating ubiquitination is critical in innate immunity. However, many viral proteins manipulate the UPS to inhibit the IFN system. Influenza A virus (IAV) NS1 inhibits RIG-I K63-linked ubiquitination by interacting with TRIM25 (55). Rotavirus nsp1 degrades the host antiviral proteins by hijacking the cullin-RING E3 Ub ligases, which inhibits the IFN system (56). Coronaviruses also have evolved mechanisms of manipulating the UPS to degrade immune-related proteins (57, 58). This immune evasion strategy can provide more time for coronaviruses to replicate, thereby increasing their survival and spreading ability (58). The present study demonstrated that SADS-CoV nsp1 induced JAK1 and CBP degradation through the Ub-proteasome pathway, suggesting that nsp1 blocked the host IFN system by hijacking the UPS (Fig. 9). Nevertheless, the specific mechanisms underlying this interesting finding remain unclear. It is believed that there are two potential mechanisms: SADS-CoV nsp1 might interact with E3 Ub ligase to manipulate UPS (as influenza A virus NS1 does) or directly act as an E3 Ub ligase to induce the ubiquitination of target proteins (as rotavirus nsp1 does). Resolving this question would improve understanding of the molecular mechanisms of SADS-CoV infection.

Overall, this study indicated that SADS-CoV nsp1 inhibited the phosphorylation of STAT1 by inducing JAK1 degradation through K11/K48-linked JAK1 polyubiquitination. Furthermore, nsp1 blocked p-STAT1 acetylation and dephosphorylation by inducing CBP degradation. These findings increase our knowledge of SADS-CoV immune evasion strategies and enhance comprehension of the mechanism implicated in the coronavirus nsp1's capacity to inhibit the IFN response.

## Experimental procedures

### Virus, cells, and plasmids

SADS-CoV (MK994934.1), SADS-CoV-mutant (the SADS-CoV nsp1 Phe39 and Leu98 were mutated to alanine), and SeV were stored in the authors’ laboratory. HEK-293T cells, Vero cells, ST cells, and LLC-PK1 cells were cultured in DMEM containing 10% fetal bovine serum, 1% penicillin, and streptomycin. The pCAGGS-3×Flag-nsp1, pCAGGS-3×Flag-nsp1-mutant, pCAGGS-2×HA-STAT1, pCAGGS-2×HA-STAT2, pCAGGS-2×HA-IRF9, pIFN-β-luc, pRL-TK, and the plasmids expressing Ub and Ub mutants were stored in the authors’ laboratory.

### Antibodies and reagents

This study used the following antibodies and reagents: mouse anti-FLAG-tag monoclonal antibody (mAb), mouse anti-HA-tag mAb, horseradish peroxidase (HRP)-conjugated goat anti-mouse (H+L), HRP-conjugated goat anti-rabbit (H+L), HRP-conjugated mouse anti-rabbit (L), HRP-conjugated goat anti-mouse (L), p-STAT1 rabbit mAb, STAT1 rabbit mAb, p-STAT2 rabbit mAb, STAT2 rabbit mAb, IRF9 rabbit mAb, p-JAK1 rabbit mAb, JAK1 rabbit mAb, TYK2 rabbit mAb, and p-TYK2 rabbit mAb (ABclonal); CREB-BP rabbit mAb (Affinity); beta-actin rabbit antibody (Proteintech); MG132 and Z-VAD-FMK (Beyotime); CHX710 (MedChemExpress); human IFN-β (InvivoGen); Lipofectamine 3000 transfection reagent (Sigma); and Dylight-conjugated 488 goat anti-mouse IgG (Abbkine). SADS-CoV-N mouse polyclonal antibodies (pAb) were stored in the authors’ laboratory.

### Virus growth kinetics

Vero cells and ST cells were cultured in 12-well plates. The cells were infected with SADS-CoV-WT or SADS-CoV-mutant at a multiplicity of infection of 0.1. The medium was changed after 2 hpi. Then, the infected cell supernatants were collected at 6, 8, 12, 16, 24, and 32 hpi and titrated on Vero cells. The virus titers were determined by calculating the log_10_TCID_50_ (median tissue culture infective dose)/ml using the Reed-Muench method.

### Immunofluorescence assay

Vero cells were infected with SADS-CoV-WT or SADS-CoV-mutant at a multiplicity of infection of 0.1 for 12 h. The cells were washed three times using phosphate-buffered saline, fixed in 4% paraformaldehyde, permeabilized with 0.1% Triton X-100, and blocked with 2% bovine serum albumin. The cells were incubated with primary antibody overnight at 4 °C, then incubated with secondary antibody for 2 h at 37 °C. The nucleus was stained with DAPI. The percentage of the number of infected cells (green) to the total number of cells (blue) was determined using ImageJ. The statistical experiments were repeated three times.

### Relative quantitative real-time PCR

HEK-293T cells (1000 ng per well in 12-well plates) were transfected with pCAGGS-2×HA-STAT1/pCAGGS-2×HA-STAT2/pCAGGS-2×HA-IRF9/ISGF3 (pCAGGS-2×HA-STAT1, pCAGGS-2×HA-STAT2, and pCAGGS-2×HA-IRF9). After 12 h, the cells (500 ng per well) were transfected with pCAGGS-3×Flag-nsp1. Then, the cells were collected, and the RNA was extracted using an RNA Easy Fast Tissue/Cell Kit (Tiangen). Genomic DNA was eliminated from the RNA by DNase I treatment, followed by complementary DNA synthesis using reverse transcription–PCR according to the manufacturer’s guidelines. The ISG15, ISG56, and OAS1 expression levels were analyzed with relative quantitative real-time PCR using the LineGene9600 RT-PCR system.

### Luciferase reporter gene assay

HEK-293T cells were cotransfected with 500 ng plasmids expressing STAT1/STAT2/IRF9/ISGF3 (pCAGGS-2×HA-STAT1, pCAGGS-2×HA-STAT2, and pCAGGS-2×HA-IRF9), 100 ng pIFN-β-luc, and 25 ng pRL-TK. After 12 h, cells were transfected with pCAGGS-3×Flag-nsp1 (200 ng per well) in 24-well plates. After 12 h, the cells were collected to detect ISRE promoter activity through the dual luciferase reporter assay.

### Western blotting

HEK-293T cells and LLC-PK1 cells were transfected with pCAGGS-3×Flag-nsp1. After 24 h, the HEK-293T cells were incubated with human IFN-β for 4 h, and the LLC-PK1 cells were stimulated by SeV. Then, the cells were collected using a lysis buffer containing 20 mM Tris (pH 7.5), 150 mM NaCl, 1 mM EDTA, 1 mM phenylmethanesulfonyl fluoride, 0.1% sodium dodecyl sulfate (SDS), 1% Triton X-100, and 0.5% sodium deoxycholate. Subsequently, the samples were treated with phosphorylation inhibitors for 30 min. The samples were mixed with loading buffer containing β-mercaptoethanol and heated at 100 °C for 10 min. The samples were separated by SDS-PAGE (polyacrylamide gel electrophoresis) and transferred to PVDF membranes for immunoblotting. The membranes were examined with the primary antibodies at 4 °C overnight and then exposed to the secondary antibodies for 1 h at room temperature. The bands were visualized using the Tanon imaging system and examined with ImageJ.

### Co-immunoprecipitation assay

HEK-293T cells were transfected with pCAGGS-2×HA-Ub or a plasmid panel expressing Ub mutants. After 12 h, the cells were transfected with pCAGGS-3×Flag-nsp1. The cells were collected using IP buffer [containing 20 mM Tris (pH 7.5), 150 mM NaCl, 1 mM EDTA, and 1% TritonX-100] for 30 min. The cell lysates were incubated overnight at 4 °C with anti-HA beads. After three washes, the beads were supplemented with the SDS loading buffer and incubated at 95 °C for 5 min. The protein expression levels were analyzed using Western blotting.

### Statistical analysis

Statistical analyses were conducted using Student’s *t* test to analyze two groups of data. All differences were considered statistically significant when *p* < 0.05, *p* < 0.01, and *p* < 0.001.

## Data availability

All data are available from the corresponding author upon a reasonable request.

## Conflict of interest

The authors declare no conflict of interest with the contents of this article.
